# Hematoma Ventricle Distance on Computed Tomography Predicts Poor Outcome in Intracerebral Hemorrhage

**DOI:** 10.3389/fnins.2020.589050

**Published:** 2020-11-19

**Authors:** Lan Deng, Yun-Dong Zhang, Jian-Wen Ji, Wen-Song Yang, Xiao Wei, Yi-Qing Shen, Rui Li, Shu-Qiang Zhang, Xin-Ni Lv, Xin-Hui Li, Zhou-Ping Tang, Guo-Feng Wu, Li-Bo Zhao, Peng Xie, Qi Li

**Affiliations:** ^1^Department of Neurology, The First Affiliated Hospital of Chongqing Medical University, Chongqing, China; ^2^Department of Neurology and Neurosurgery, The Third Affiliated Hospital of Chongqing Medical University, Chongqing, China; ^3^NHC Key Laboratory of Diagnosis and Treatment on Brain Functional Diseases, The First Affiliated Hospital of Chongqing Medical University, Chongqing, China; ^4^Department of Medical Technology, Chongqing Medical and Pharmaceutical College, Chongqing, China; ^5^Department of Neurology, Tongji Hospital, Tongji Medical College, Huazhong University of Science and Technology, Wuhan, China; ^6^Emergency Department, The Affiliated Hospital of Guizhou Medical University, Guiyang, China; ^7^Chongqing Key Laboratory of Cerebrovascular Disease Research, Yongchuan Hospital of Chongqing Medical University, Chongqing, China; ^8^Department of Neurology, Yongchuan Hospital of Chongqing Medical University, Chongqing, China

**Keywords:** intracerebral haemorrhage, hematoma ventricle distance, outcome, neuroimaging, stroke

## Abstract

**Objective:**

To investigate the relationship between hematoma ventricle distance (HVD) and clinical outcome in patients with intracerebral hemorrhage (ICH).

**Methods:**

We prospectively enrolled consecutive patients with ICH in a tertiary academic hospital between July 2011 and April 2018. We retrospectively reviewed images for all patients receiving a computed tomography (CT) within 6 h after onset of symptoms and at least one follow-up CT scan within 36 h. The minimum distance of hematoma border to nearest ventricle was measured as HVD. Youden index was used to evaluate the cutoff of HVD predicting functional outcome. Logistic regression model was used to assess the HVD data and clinical poor outcome (modified Rankin Scale 4–6) at 90 days.

**Results:**

A total of 325 patients were included in our final analysis. The median HVD was 2.4 mm (interquartile range, 0–5.7 mm), and 119 (36.6%) patients had poor functional outcome at 3 months. After adjusting for age, admission Glasgow coma scale, intraventricular hemorrhage, baseline ICH volume, admission systolic blood pressure, blood glucose, hematoma expansion, withdrawal of care, and hypertension, HVD ≤ 2.5 mm was associated with increased odds of clinical poor outcome [odd ratio, 3.59, (95%CI = 1.72–7.50); *p* = 0.001] in multivariable logistic regression analysis.

**Conclusion:**

Hematoma ventricle distance allows physicians to quickly select and stratify patients in clinical trials and thereby serve as a novel and useful addition to predict ICH prognosis.

## Introduction

Intracerebral hemorrhage (ICH) is the most severe and devastating stroke subtype, with an up to 40% mortality at 30 days (van Asch et al., 2010; Krishnamurthi et al., 2014; Poon et al., 2014). Only 12–39% of ICH survivors achieve long-term functional independence (Krishnamurthi et al., 2014; An et al., 2017). Baseline hematoma volume (Broderick et al., 1993), infratentorial location (Delcourt et al., 2017), and the presence of intraventricular hemorrhage (Witsch et al., 2015; IVH) are independent predictors of functional outcome. Among them, the hematoma volume is the most powerful predictor of poor functional outcome. Several imaging markers such as computed tomographic (CT) angiography spot sign and non-contrast CT markers are also predictive of outcome (Sporns et al., 2018).

The location of hematoma also plays a key role in predicting outcome. Recent studies identified several affected anatomic regions that were associated with functional outcomes (Eslami et al., 2019). It is reported hematomas that involve the posterior limb of internal capsule or thalamus increases the risks of death or disability and of disability alone. However, few studies focused on specific brain structures and functional outcome. We hypothesized that hematomas located in the deep brain structures or close to the ventricles are more likely to have worse functional outcome than superficially located hematomas. We aim to derive a location-specific outcome prediction model to enhance the clinicians’ ability to prognosticate functional outcome after ICH. We hypothesized that patients may have poor outcome if the hematoma disrupts deep brain structures. Ventricles are located in the deep brain area, and the distance from the hematoma border to the ventricle may reflect the extent of involvement of deep brain structures.

Therefore, we derived a simple CT-based measurement of hematoma ventricle distance (HVD) to reflect the distance of hematoma to ventricles. The aim of our study was to investigate the association between HVD and functional outcome.

## Materials and Methods

We prospectively enrolled consecutive patients with spontaneous non-lobar ICH in a tertiary academic hospital between July 2011 and April 2018. Briefly, we included patients with age > 18 years old, who received a CT within 6 h after onset of symptoms and at least one follow-up CT scan within 36 h. The main exclusion criteria were (1) primary IVH; (2) anticoagulants-associated bleeding; (3) secondary ICH due to trauma or tumor; (4) lobar hemorrhage; (5) secondary ICH due to rupture of arteriovenous malformation or intracranial aneurysm; and (6) surgery prior to follow-up CT.

Enrollment and data collection have been previously described (Li et al., 2019, 2020). In brief, the baseline clinical data included demographic data, hypertension, medication use, admission blood pressure, the National Institute of Health Stroke Scale (NIHSS) score, Glasgow Coma Scale (GCS) score, and pre-morbid modified Rankin Scale (mRS). End point was assessed using mRS score at 90 days. Poor outcome was defined as a mRS score of 4 to 6 (Eslami et al., 2019). Written informed consent was obtained from patients or legal representatives before enrollment. Approval for this study was obtained from The Ethics Committee of The First Affiliated Hospital of Chongqing Medical University.

**FIGURE 1:**
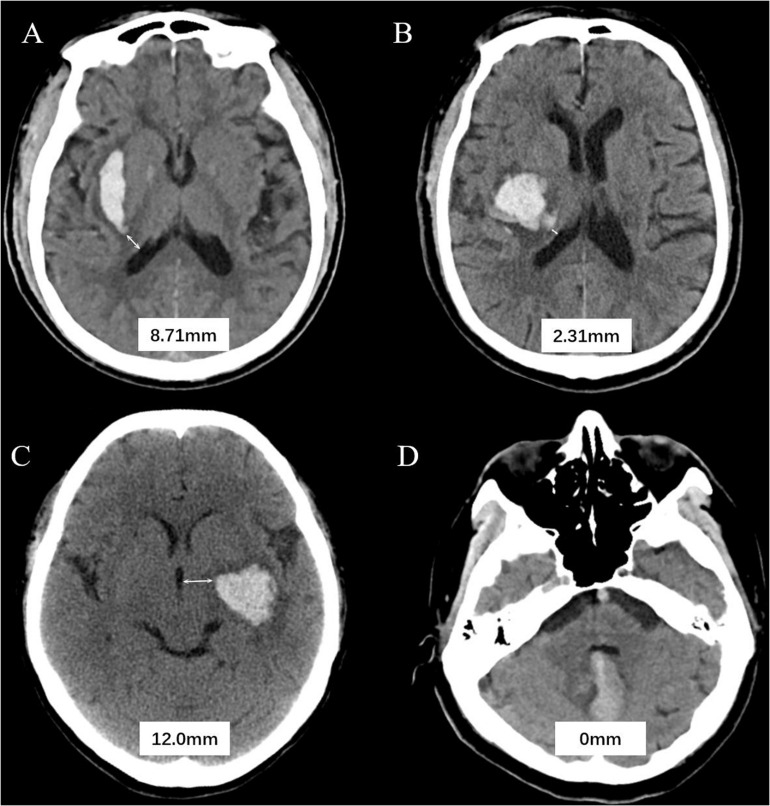
Graphic representation of the hematoma ventricle distance (HVD) on computed tomography. **(A)** A Baseline CT scan reveals a basal ganglia hematoma with HVD of 8.71 mm. **(B)** In patient B, the HVD is 2.31 mm. **(C)** A admission CT shows the distance between hematoma and the third ventricle is 2.0 mm. **(D)** Cerebellar hematoma reaches the border of ventricles without IVH extension.

All CT examinations were preformed following standard protocol as described before (Li et al., 2017, 2020). CT scans with 5 mm thick slices of axial view were stored in DICOM format. Trained neurologists (blinded to clinical data and outcome) evaluated the baseline CT scans. Hematoma expansion (HE) was defined as absolute growth of >6 ml or relative growth of >33% at follow-up CT scan (Poli et al., 2020). Hematoma location was evaluated on baseline CT and classified as lobar and non-lobar ICH. Lobar hemorrhage was defined as involvement of cortex or cortical–subcortical junction hematoma. Hematomas that are located at thalamus, basal ganglia, internal capsule, deep periventricular white matter, brain stem, or cerebellum was defined as non-lobar hemorrhage (Morotti et al., 2020). HVD was defined as minimum distance of hematoma border to nearest ventricle (Figure 1). HVD was measured independently by two trained researchers who were blinded to the clinical profiles of the patients, and HVD was measured using RadiAnt DICOM Viewer (Version 2020.2; Medixant Corporation, Poznań, Poland). The HVD was 0 if the hematoma reaches the border of ventricles with and without IVH extension (Figure 1D). We have operationally defined deeply located ICH as hematomas with HVD ≤ 2.5 mm.

### Statistical Analyses

All Statistical analyses were performed using SPSS software (Version 23.0; IBM Corporation, Armonk, NY, United States). Continuous variables were presented by mean [standard deviation (SD)] or median [interquartile range (IQR)], and categorical variables were summarized using number (percentage). Intergroup differences were assessed using Fisher exact test, Student’s *t* test, or the Mann–Whitney *U* test as appropriate. Intra-class correlation coefficient (ICC) was used to determine the interobserver agreement. Receiver operating characteristic curves were used to determine HVD cutoffs and associated sensitivity and specificity in predicting 3-month poor outcome. The Youden Index combining sensitivity and specificity was used for classification of dichotomous outcome to identify the cutoff of HVD. Then, the identified cutoffs were validated by univariable logistic regression model and multivariable logistic regression models. Since some of the variables are related to each other (e.g., HVD and neuroimaging variables), variance inflation factor (VIF) was used to calculate the collinearity, and a predictor of VIF > 3 was considered as an indicative collinearity. Multivariable logistic regression model was built using covariates with *p* < 0.1 in univariable logistic regression. We conducted a stepwise forward multivariable logistic regression model to assess whether deeply located ICH (HVD ≤ 2.5 mm), age, hypertension history, admission diastolic blood pressure, baseline hematoma volume, HE, blood glucose, withdrawal of care, and presence of IVH were associated with poor clinical outcome at 90 days. All selected variables were presented with odds ratio (OR), 95% confidence interval (CI), and *p* value. The *p* value of score test was displayed in non-selected variables, and no interaction terms were shown. We ascertained the calibration of the model using likelihood ratio and Hosmer and Lemeshow goodness-of-fit test. A *p* value < 0.05 was considered for statistical significance.

## Results

**FIGURE 2:**
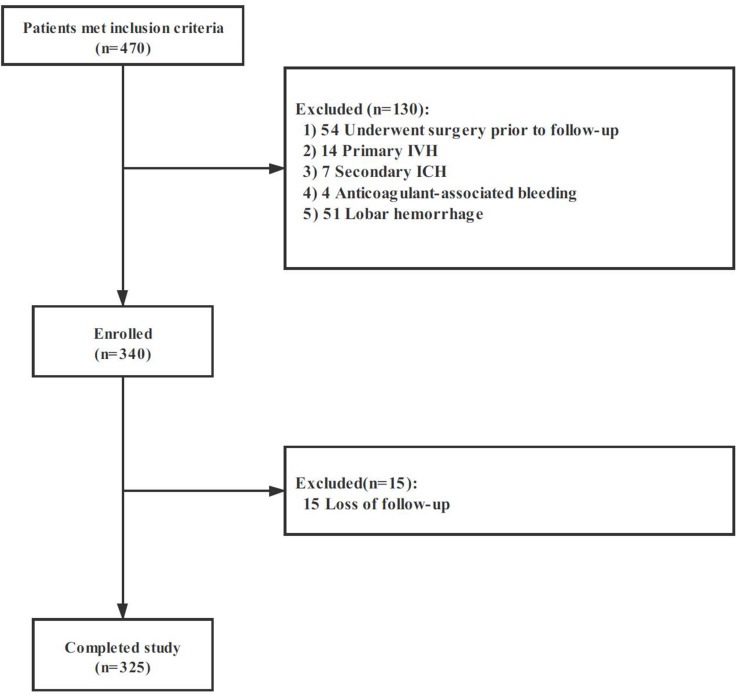
Cohort selection flowchart. Abbreviation: IVH, intraventricular hemorrhage; ICH, intracerebral hemorrhage.

Among 470 spontaneous ICH, 325 patients met the eligibility criteria and were included in our study. The flow diagram of patient selection is illustrated in Figure 2. The study population included 209 (64.3%) men, and IVH extension was observed in 104 of 325 (32.0%) patients on the initial CT scan. Mean (±SD) ICH volume was 16.5 ± 14.0 ml. The ICH lesions were in the basal ganglia (*n* = 213, 65.5%), thalamus (*n* = 84, 25.9%), brainstem (*n* = 11, 3.4%), and cerebellum (*n* = 17, 5.2%). A total of 119 participants (36.6%) with poor clinical outcome had worse clinical severity scores (GCS and NIHSS, both *p* < 0.001), larger baseline hematoma volumes (*p* < 0.001), more IVH (*p* < 0.001), and more likely to be older (*p* = 0.008). The detailed intergroup differences between participants with poor clinical outcome and those without are shown in Table 1.

**TABLE 1:** Comparison of baseline demographic, clinical, and radiological characteristics between patients with and without poor outcome.

**Variables**	**Poor outcome (*n* = 119)**	**Favorable outcome (*n* = 206)**	***p* value**
**Demographic**			
Mean age, years (SD)	61.6 (12.3)	57.9 (11.9)	0.008
Sex, male, *n* (%)	79 (66.4)	130 (63.1)	0.552
**Medical history**			
Alcohol consumption, *n* (%)	48 (40.3)	83 (40.3)	0.994
Smoking, *n* (%)	56 (52.4)	87 (42.2)	0.398
Hypertension, *n* (%)	93 (78.2)	141 (68.4)	0.060
Diabetes mellitus, *n* (%)	12 (10.1)	19 (9.2)	0.799
**Clinical features**			
Systolic blood pressure, mmHg (SD)	177.8 (31.8)	169.3 (26.0)	0.014
Diastolic blood pressure, mmHg (SD)	102.2 (20.2)	98.9 (15.4)	0.136
Admission GCS score, median (IQR)	11.0 [7.0–14.0]	14.0 [13.0–15.0]	<0.001
Admission NIHSS score, median (IQR)	18.0 [11.0–26.0]	8.0 [4.0–12.0]	<0.001
Time from onset to CT, h IQR)	2.0 [1.0–3.0]	2.0 [1.0–4.0]	0.212
Withdrawal of care, *n* (%)	32 (26.9)	3 (1.5)	<0.001
Blood glucose, (mmol/L)	7.4 [6.2–9.3]	6.3 [5.4–7.5]	<0.001
**Radiological characteristics**			
Baseline ICH volume, ml (IQR)	18.6 [11.8–31.5]	10.5 [6.5–15.8]	<0.001
IVH at baseline CT, *n* (%)	58 (48.7)	46 (22.3)	<0.001
SAH at baseline CT, *n* (%)	11 (5.4)	6 (5.0)	0.900
HVD, mm (IQR)	0 [0–2.8]	4.4 [0–6.7]	<0.001
Hematoma expansion, *n* (%)	52 (43.7)	34 (16.5)	<0.001
HVD ≤ 2.5 mm, *n* (%)	88 (73.9)	79 (38.3)	<0.001

*Abbreviations: ICH, intracerebral hemorrhage; CT, computed tomography; IVH, intraventricular hemorrhage; SAH, subarachnoid hemorrhage; GCS, Glasgow Coma Scale; NIHSS, National Institutes of Health Stroke Scale; HVD, hematoma ventricle distance; IQR, inter-quartile range; and SD, standard deviation.*

Excellent interobserver agreement [ICC = 0.882 (95%CI, 0.798–0.935), *p* < 0.001] and intrarater agreement [ICC = 0.941 (95%CI, 0.926–0.953), *p* < 0.001] were obtained for measurement of HVD. Overall, the median HVD was 2.4 (IQR, 0–5.7) mm. Patients with poor clinical outcome were more likely to have deeply located ICH (median 0 mm vs. 4.4 mm, *p* < 0.001, Table 1). We identified an optimal cutoff value of 2.47 mm for predicting poor outcome, and 2.5 mm was operationally chosen as clinically useful cutoff value. On baseline CT, deeply located ICH (HVD ≤ 2.5 mm) was observed in 167 (51.4%) participants. Among those, 104 out of 167 (62.3%) had concurrent IVH. In patients with deeply located ICH (HVD ≤ 2.5 mm), these were located in basal ganglia (*n* = 71, 42.5%), thalamus (*n* = 76, 45.5%), brainstem (*n* = 7, 4.2%), and cerebellum (*n* = 13, 7.8%). Patients with deeply located ICH (HVD ≤ 2.5 mm) were more likely to have higher NIHSS score (*p* < 0.001), lower GCS score (*p* < 0.001) and poor outcome (*p* < 0.001; Table 2).

**TABLE 2:** Comparison of baseline demographic, clinical, and radiological characteristics between patients with hematoma ventricular distance shorter than 2.5 mm and those without.

**Variables**	**HVD ≤ 2.5 mm (*n* = 167)**	**HVD>2.5 mm (*n* = 158)**	***p* value**
**Demographic**			
Mean age, years (SD)	60.1 (12.2)	58.5 (12.1)	0.234
Sex, male, *n* (%)	103 (61.7)	106 (67.1)	0.309
**Medical history**			
Alcohol consumption, *n* (%)	63 (37.7)	68 (43.0)	0.329
Smoking, *n* (%)	73 (43.7)	70 (44.3)	0.915
Hypertension, *n* (%)	126 (75.4)	108 (68.4)	0.155
Diabetes mellitus, *n* (%)	19 (11.4)	12 (7.6)	0.246
**Clinical features**			
Systolic blood pressure, mmHg (SD)	174.9 (30.3)	169.8 (26.4)	0.112
Diastolic blood pressure, mmHg (SD)	100.6 (19.5)	99.6 (15.7)	0.597
Admission GCS score, median (IQR)	13.0 [9.0–14.0]	14.0 [13.0–15.0]	<0.001
Admission NIHSS score, median (IQR)	13.0 [8.0–23.0]	9.0 [5.0–13.3]	<0.001
Time from onset to CT, h (IQR)	2.0 [1.0–3.0]	2.0 [1.0–4.0]	0.351
Withdrawal of care, *n* (%)	27 (16.2)	8 (5.1)	0.001
Blood glucose, (mmol/L)	7.0 [5.9–8.8]	6.2 [5.3–7.4]	<0.001
**Radiological characteristics**			
Baseline ICH volume, ml (IQR)	13.4 [8.0–21.7]	12.1 [7.3–19.3]	0.090
IVH at baseline CT, *n* (%)	104 (62.3)	0 (0)	<0.001
SAH at baseline CT, *n* (%)	11 (6.6)	6 (3.8)	0.254
Hematoma expansion, *n* (%)	44 (26.3)	42 (26.6)	0.962
Outcome	15 (10.7)	13 (10.3)	<0.001
90-day mRS, mm (IQR)	4.0 [1.0–5.0]	2.0 [1.0–3.0]	<0.001
Poor outcome, *n* (%)	88 (52.7)	31 (19.6)	<0.001
Mortality at 90 days, *n* (%)	40 (24.0)	11 (7.0)	<0.001

*Abbreviations: ICH, intracerebral hemorrhage; CT, computed tomography; IVH, intraventricular hemorrhage; SAH, subarachnoid hemorrhage; GCS, Glasgow Coma Scale; NIHSS, National Institutes of Health Stroke Scale; HVD, hematoma ventricle distance; mRS, modified Rankin scale; IQR, inter-quartile range; and SD, standard deviation.*

**FIGURE 3:**
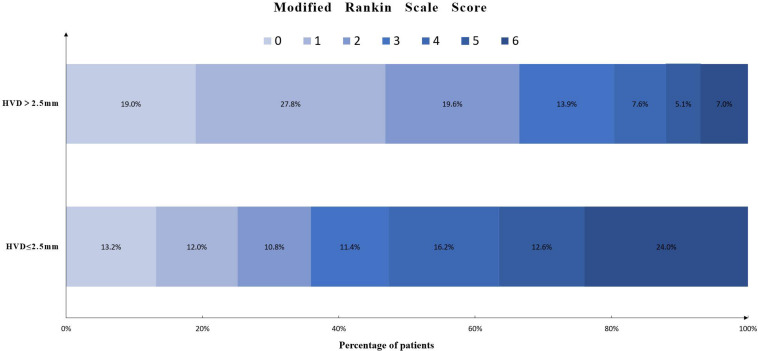
Distribution of modified Rankin scale score in patients with HVD ≤ 2.5 mm or HVD > 2.5 mm. Abbreviation: HVD, hematoma ventricle distance.

The relationship between HVD and 90-day mRS score is shown in Figure 3. A total of 119 (36.6%) had poor functional outcome (mRS = 4–6) at 3-month follow-up. Patients with deeply located ICH (HVD ≤ 2.5 mm) were associated with poor functional outcome [OR, 3.59 (95%CI = 1.72–7.50); *p* = 0.001].

Univariate logistic regression showed that older age, higher systolic blood pressure, lower GCS score, higher NIHSS score, larger baseline hematoma volume, higher blood glucose, hematoma expansion, withdrawal of care, presence of IVH as well as the HVD increased the odds of poor clinical outcome (Table 3). In multivariable logistic regression analysis, HVD ≤ 2.5 mm remained an independent predictor of functional independence [OR, 3.59 (95%CI = 1.72–7.50); *p* = 0.001; Table 3] after adjusting for age, admission GCS score, systolic blood pressure, hypertension, baseline ICH volume, blood glucose, hematoma expansion, withdrawal of care, and IVH. This model was well built (likelihood ratio test, *p* < 0.001), and Hosmer and Lemeshow goodness-of-fit test showed good calibration (χ^2^ = 2.4; *p* = 0.967).

**TABLE 3:** Univariate and multivariate analysis of predictors for poor outcome (mRS 4–6) at 3 months.

**Variable**	**Univariate**		**Multivariate**	
	**OR (95% CI)**	***p* value**	**OR (95% CI)**	***p* value**
Age, year^a^	1.03 (1.01–1.05)	0.009	1.04 (1.01–1.07)	0.027
Gender	1.12 (0.72–1.86)	0.552		
Admission GCS score^a^	0.72 (0.66–0.78)	<0.001	0.78 (0.70–0.88)	<0.001
Admission NIHSS score^a^	1.13 (1.10–1.17)	<0.001		
Admission SBP, mmHg^a^	1.01 (1.00–1.02)	0.005	…	NS:0.338
Time from onset to CT, h^a^	0.90 (0.78–1.04)	0.148		
Alcohol consumption	1.00 (0.63–1.59)	0.994		
Smoking	1.22 (0.77–1.91)	0.399		
Hypertension	1.65 (0.98–2.79)	0.062	…	NS:0.648
Blood glucose, mmol/L	1.26 (1.13–1.42)	<0.001	…	NS:0.173
Baseline ICH volume, ml^a^	1.08 (1.05–1.10)	<0.001	1.07 (1.03–1.10)	<0.001
SAH at baseline CT	0.94 (0.34–2.60)	0.900		
IVH at baseline CT	3.31 (2.03–5.38)	<0.001	…	NS:0.231
Hematoma expansion	3.93 (2.34–6.58)	<0.001	3.61 (1.64–7.96)	0.001
Withdrawal of care	24.89 (7.42–83.448)	<0.001	22.82 (2.58–201.15)	0.005
HVD	0.81 (0.75–0.88)	<0.001		
HVD ≤ 2.5 mm	4.56 (2.78–7.50)	<0.001	3.59 (1.72–7.50)	0.001

*Multivariable analysis was used stepwise forward selection. All selected variables were presented with OR, 95%CI, and *p* value. The *p* value of score test was displayed in non-selected variables, and no interaction terms were shown. Abbreviations: mRS, modified Rankin scale; ICH, intracerebral hemorrhage; CT, computed tomography; SBP, systolic blood pressure; IVH, intraventricular hemorrhage; SAH, subarachnoid hemorrhage; GCS, Glasgow Coma Scale; NIHSS, National Institutes of Health Stroke Scale; HVD, hematoma ventricle distance; OR, odds ratio; CI, confidence interval; and NS, not selected. ^*a*^Per unit change in regressor.*

In the receiver operating characteristic analysis for the poor clinical outcome (mRS 3–6, without cerebellar/brainstem hemorrhage), the area under the curve of deeply located ICH (HVD ≤ 2.5 mm), HVD, and IVH were 0.69, 0.67, and 0.63, respectively. The sensitivity, specificity, positive predictive value, and negative predictive value of HVD ≤ 2.5 mm for predicting poor clinical outcome at 3 months were 65.5, 66.4, 66.0, and 66.0%, respectively.

## Discussion

In our study, we demonstrated that the distance between ventricle and hematoma on CT was independently associated with poor clinical outcome in patients with ICH. Furthermore, HVD seemed to be a better predictor of functional outcome as compared with baseline IVH. Our study provided evidence that deeply located ICHs (HVD ≤ 2.5 mm) were associated with poor functional outcome in patients with ICH. Since CT is widely available in almost all clinical settings, our finding may allow physicians to quickly select and stratify patients in clinical trials.

Non-contrast CT is a widely available diagnostic method of choice in patients with ICH. In our study, we have proposed a novel parameter called “hematoma ventricle distance” that represents the anatomic location of hematoma to ventricles. Previous studies suggested that deep and lobar ICHs may have different pathophysiological features (Hanley, 2009; Falcone et al., 2013). Basal ganglia or thalamic hemorrhages are more likely to damage deep brain structure than lobar hemorrhages. Non-lobar hemorrhages are more likely caused by hypertension, while lobar hemorrhages are mostly cerebral amyloid angiopathy related (Matsukawa et al., 2012; Falcone et al., 2013). Therefore, lobar hemorrhages were excluded. We have shown that the parameter is easy to measure with good inter-rater agreement, and the HVD is easy to identify on the baseline CT scan in comparison to other prognostic parameters. To our knowledge, this is the first study that demonstrated the predictive value of hematoma-to-ventricle distance with clinical outcome. Further, we investigated the quantitative threshold of HVD in predicting favorable and poor clinical outcome and found that a value of 2.5 mm could be optimal. Multivariable logistic regression analysis showed deeply located ICH (HVD ≤ 2.5 mm), GCS score, baseline ICH volume, HE, and withdrawal of care were independent predictors of functional independence.

The location of hematoma and functional outcome has been explored in several studies (Arboix et al., 2002; Delcourt et al., 2017; Eslami et al., 2019). A larger study of 2,066 patients from Intensive Blood Pressure Reduction in Acute Cerebral Haemorrhage Trial 2 (INTERACT 2) suggested that posterior limb of internal capsule, thalamus, and infratentorial involvement were associated with poor outcome (Lee et al., 2015; Delcourt et al., 2017). A recent secondary analysis of 467 ICH patients from Clot Lysis: Evaluating Accelerated Resolution of Intraventricular Hemorrhage III trial (CLEAR III) showed thalamic hemorrhage globus pallidus/putamen and posterior limb internal capsule had the associations with worse clinical outcome (Eslami et al., 2019). Another set of 1345 patients from Ethnic/Racial Variations of ICH (ERICH) study also reported poor outcome for thalamic hemorrhage (Leasure et al., 2018). These studies investigated the relationship between the specific anatomic hematoma location and clinical outcome, and some studies indicated the hematomas that are close to ventricles are more likely to have poor outcome (Feng et al., 2020). However, they did not further confirm it. We investigated the distance between hematoma margin and ventricle system, and it suggested that deeply located ICH (HVD ≤ 2.5 mm) represented a subtly different neuroimaging feature.

We also found a significant proportion of patients with HVD ≤ 2.5 mm had concurrent ICH. Recent studies suggested that IVH was a powerful predictor of poor clinical outcome, and reported 30-day mortality rate for patients with IVH was nearly 5 times higher than those without IVH (Tuhrim et al., 1999; Steiner et al., 2006; Mayer et al., 2008; Hanley, 2009; Hinson et al., 2010). In our study, IVH was an independent predictor of poor outcome in univariate analysis. We did not find IVH to be an independent predictor of poor clinical outcome in multivariate model (*p* = 0.139). However, HVD ≤ 2.5 mm remains an independent predictor of poor outcome in the multivariable logistic regression model, suggesting that HVD ≤ 2.5 mm is a more robust outcome parameter than IVH presence. We also demonstrated that HVD ≤ 2.5 mm has relatively high sensitivity for predicting poor outcome. Therefore, it might be used as an easy-to-use imaging marker for screening of patients who were likely to have poor outcome. However, future studies are needed to explore whether these patients are potential candidates for surgical or interventions.

The exact underlying mechanism of HVD and outcome remains unknown. A possible explanation is that hematomas that are close to ventricles are more likely to have short HVD. Recent studies suggested that structural lesions in the periventricular areas may cause clinical symptoms because of disruption of fiber tracts (Hanley, 2009; Eslami et al., 2019; Hurford et al., 2019). The clinical severity of the deficit is dependent on the anatomic location of such lesions and the functional integrity of affected fiber bundles. Since the periventricular area is located deep within the brain where white matter tracts are abundant, damage to this area is highly likely to have severe neurological deficits. Therefore, future studies employing diffuse tensor imaging of white matter tract damages are needed to validate this hypothesis.

There are several limitations in our study. First, our study is a retrospective analysis of a single-center cohort. Second, the sample size was relatively small, and it was not externally validated. Third, we only included patients with presumed hypertensive ICH. Whether our findings are generalizable to patients with lobar ICH is unclear. Fourth, computed tomography angiography (CTA) spot sign was not compared with HVD to see if alone or in combination they could give us better predictive power.

In summary, we demonstrated HVD ≤ 2.5 mm was associated with poor outcome independent of other established predictors. HVD seems to be an easy-to-use parameter for prognostic stratification in clinical settings, which could help guide the use of clinical resources by identifying patients who might benefit from intensive care.

## Data Availability Statement

The datasets presented in this article are not readily available because the datasets generated for this study are available on request to the corresponding author. Requests to access the datasets should be directed to QL qili_md@126.com.

## Ethics Statement

The studies involving human participants were reviewed and approved by The Ethics Committee of The First Affiliated Hospital of Chongqing Medical University. The patients/participants provided their written informed consent to participate in this study.

## Author Contributions

QL and LD: study concept and design. QL, RL, L-BZ, Y-DZ, J-WJ, W-SY, Y-QS, LD, X-NL, XW, and S-QZ: acquisition of data. LD: statistical analysis. Analysis and interpretation of data: all authors. LD: drafting of the manuscript. QL, Y-DZ, J-WJ, G-FW, Z-PT, and PX: critical revision of the manuscript for important intellectual content. QL: obtained funding. QL, Y-DZ, and PX: study supervision.

## Conflict of Interest

The authors declare that the research was conducted in the absence of any commercial or financial relationships that could be construed as a potential conflict of interest.
